# Three Pathogens in Sympatric Populations of Pumas, Bobcats, and Domestic Cats: Implications for Infectious Disease Transmission

**DOI:** 10.1371/journal.pone.0031403

**Published:** 2012-02-08

**Authors:** Sarah N. Bevins, Scott Carver, Erin E. Boydston, Lisa M. Lyren, Mat Alldredge, Kenneth A. Logan, Seth P. D. Riley, Robert N. Fisher, T. Winston Vickers, Walter Boyce, Mo Salman, Michael R. Lappin, Kevin R. Crooks, Sue VandeWoude

**Affiliations:** 1 USDA National Wildlife Disease Program, Fort Collins, Colorado, United States of America; 2 Department of Microbiology, Immunology, and Pathology, Colorado State University, Fort Collins, Colorado, United States of America; 3 U.S. Geological Survey, Western Ecological Research Center, Thousand Oaks, California, United States of America; 4 Colorado Parks and Wildlife, Fort Collins, Colorado, United States of America; 5 Colorado Parks and Wildlife, Montrose, Colorado, United States of America; 6 National Park Service, Thousand Oaks, California, United States of America; 7 Wildlife Health Center, University of California Davis, Davis, California, United States of America; 8 Department of Clinical Sciences, Colorado State University, Fort Collins, Colorado, United States of America; 9 Department of Fish, Wildlife, and Conservation Biology, Colorado State University, Fort Collins, Colorado, United States of America; 10 U.S. Geological Survey, Western Ecological Research Center, San Diego, California, United States of America; University of Georgia, United States of America

## Abstract

Anthropogenic landscape change can lead to increased opportunities for pathogen transmission between domestic and non-domestic animals. Pumas, bobcats, and domestic cats are sympatric in many areas of North America and share many of the same pathogens, some of which are zoonotic. We analyzed bobcat, puma, and feral domestic cat samples collected from targeted geographic areas. We examined exposure to three pathogens that are taxonomically diverse (bacterial, protozoal, viral), that incorporate multiple transmission strategies (vector-borne, environmental exposure/ingestion, and direct contact), and that vary in species-specificity. *Bartonella* spp., Feline Immunodeficiency Virus (FIV), and *Toxoplasma gondii* IgG were detected in all three species with mean respective prevalence as follows: puma 16%, 41% and 75%; bobcat 31%, 22% and 43%; domestic cat 45%, 10% and 1%. *Bartonella* spp. were highly prevalent among domestic cats in Southern California compared to other cohort groups. Feline Immunodeficiency Virus exposure was primarily associated with species and age, and was not influenced by geographic location. Pumas were more likely to be infected with FIV than bobcats, with domestic cats having the lowest infection rate. *Toxoplasma gondii s*eroprevalence was high in both pumas and bobcats across all sites; in contrast, few domestic cats were seropositive, despite the fact that feral, free ranging domestic cats were targeted in this study. Interestingly, a directly transmitted species-specific disease (FIV) was not associated with geographic location, while exposure to indirectly transmitted diseases – vector-borne for *Bartonella* spp. and ingestion of oocysts via infected prey or environmental exposure for *T. gondii* – varied significantly by site. Pathogens transmitted by direct contact may be more dependent upon individual behaviors and intra-specific encounters. Future studies will integrate host density, as well as landscape features, to better understand the mechanisms driving disease exposure and to predict zones of cross-species pathogen transmission among wild and domestic felids.

## Introduction

The effects of infectious diseases on human health have long been appreciated and their impacts on wildlife, including threatened and endangered species, are increasingly recognized [1], [2]. Zoonotic diseases are a particular concern for both human and wildlife populations, and they have been emerging worldwide with increasing frequency [3]. Disease emergence can be associated with multiple factors, but anthropogenic landscape change, often accompanied by habitat fragmentation, has played a role in several emergence events [4], [5]. In some cases, the presence of domestic animals in and around urban areas could also potentially help bridge the zoonotic infection gap that previously existed between humans and wildlife [6].

**Figure 1:**
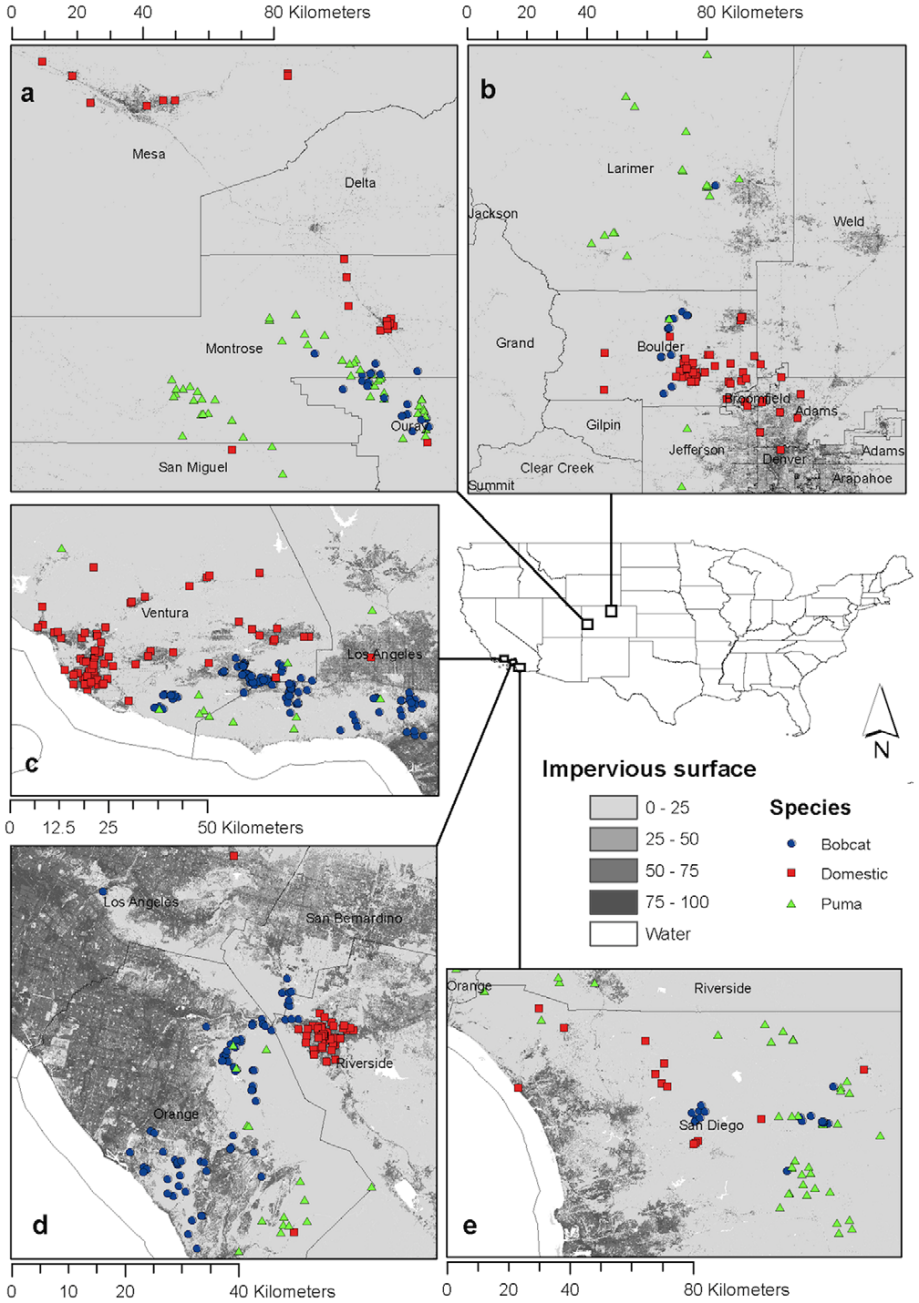
Capture locations of puma, bobcat, and domestic cat, in relation to urbanized areas, in the different study areas: (a) Colorado Western Slope, (b) Colorado Front Range, (c) Ventura County California, (d) Orange County California, and (e) Riverside/San Diego Counties, California. Impervious surface refers to artificial materials found in urban areas (asphalt, concrete, etc.) and highly compacted soils and is an indicator of urban development intensity.

In an attempt to better understand exposure to common pathogens in overlapping populations of wild and domestic animals, we examined exposure to three pathogens (two of which are zoonotic), representing different transmission modes, in three felid species: pumas (*Puma concolor*), bobcats (*Lynx rufus*), and domestic cats (*Felis domesticus*). These three species are sympatric in our study sites, especially along urban edges, and are susceptible to many of the same diseases, several of which can be transmitted both within and between species. In addition, bobcats and pumas vary in degree of contact with domestic cats, as well as in home range and resource requirements [7], [8], [9], [10]. Previous research suggested that bobcats are more likely than pumas to persist in fragmented urban habitats [11], and would be more likely to come into contact with domestic cats. These differences allow an examination of exposure to our three target pathogens, *Bartonella* spp., Feline Immunodeficiency Virus (FIV), and *Toxoplasma gondii*, across a broad range of factors. This study design allows insight into felid infectious disease transmission characteristics by using basic seroprevalence analysis on an unprecedented scale (see Figure 1).

The fundamental ecology of the three pathogens evaluated is relatively well known. *Bartonella* spp. are vector-borne bacteria known to be transmitted by *Ctenocephalides felis* fleas, although ticks and other arthropods have also been implicated as vectors [12], [13]. *Bartonella henselae*, *B. clarridgeiae*, and *B. koehlerae* commonly infect domestic cats, but infection in non-domestic felids has not been extensively characterized [14], [15]. Although domestic cats may not generally display clinical signs, even when bacteremic, human infection can cause serious disease including bacillary angiomatosis and peliosis (“cat scratch disease”). Domestic cats can influence human exposure rates by carrying *C. felis* into the home environment and by serving as a source of bacteria for *C. felis* to acquire and transmit infection to humans [15], [16]; however, the effect of infection on wild felid and feral cat health is thought to be limited [17].

Feline Immunodeficiency Virus is an enveloped RNA retrovirus and the feline analogue to human immunodeficiency virus (HIV). Each felid species is typically infected with a unique monophyletic FIV strain that is highly divergent from FIVs found in other felid species [18], supporting the hypothesis that FIV strains do not readily cross felid species boundaries. While viral genetic analyses have demonstrated that the vast majority of infections occur as a result of intra-species transmission, inter-species transmission has been documented between bobcats and pumas in California and Florida and in a small number of captive settings [18], [19]. Infection persists throughout the lifetime of the animal [20]. Infected domestic cats exhibit immunosuppression and opportunistic infections during advanced stages of the disease (generally after many years of infection). Clinical disease in non-domestic felids infected with FIV is still debated and likely is present only after several years of infection [21], [22], [23], [24], [25]. Transmission is believed to primarily occur via direct contact, especially during aggressive interactions and mating [20]. Feline Immunodeficiency Virus is significantly divergent from HIV, and zoonotic transmission of FIV to people has not been recorded [26].

*Toxoplasma gondii* is a ubiquitous protozoan parasite whose complex life-cycle culminates in passage of oocysts in feces of felids, the only known definitive hosts. The oocysts sporulate and become infectious within one to three days and can persist in soil and water for months. Oocyst ingestion is a common route of transmission for intermediate hosts that can include birds and mammals [27], [28] and while cats can be infected by ingestion of sporulated oocysts, it is believed that felids are most commonly infected by consuming infected prey that harbor the organism in muscle and other tissues [29]. Most immunocompetent felids do not suffer fitness effects from *T. gondii* infection; however, *T. gondii* is a zoonotic pathogen and serious complications can arise when vertical transmission occurs in humans during pregnancy [30] or when persons are immunosuppressed from disease or chemotherapy. Domesticated herbivores are routinely exposed to *T. gondii* via presumed ingestion of oocysts that contaminate the environment; as a result, a significant percentage of the meat supply contains infective *T. gondii* oocyts [30]. Recent research has also implicated *T. gondii* infection as a factor in declining sea otter populations on the western coast of the U.S. [31].

For the purposes of this study, exposure to each pathogen was estimated by measuring serum antibodies using previously validated assays (Table 1) and although antibody presence in serum does not always correlate with active infection or clinical disease, all three pathogens in this study have some element of chronic infections.

Because FIV infection is known to persist for the life of the animal, seropositivity would typically correlate with ongoing infection [20]. Similarly, *Bartonella* spp. infections are generally chronic, though negative blood cultures can be obtained from seropositive cats, suggesting either intermittent bacteremia or natural clearance of the infection [32]. Primary *T. gondii* in cats results in a brief period of oocyst shedding (enteroepithelial cycle) in feces. Systemic infection occurs concurrently, which ultimately leads to a chronic tissue phase of infection in which the protozoan encysts in multiple tissues, including the muscles and central nervous system of immunocompetent cats. In domestic cats, the tissue phase of infection is believed to persist for the life of the host [33]. It is unknown whether domestic cats shed oocysts more than once after natural infections; however, repeated oocyst shedding has been documented in some seropositive, experimentally infected cats after repeated exposure, during coinfection with *Isospora felis*, and under extreme immunosuppression.

**Table 1:** Comparison of the three pathogens under surveillance.

Pathogen	Class	Transmission	Clinical symptoms	Assay
*Bartonella* spp.	Bacteria	Vector-borne	Often minor, but fever, lethargy, uveitis, urinary tract disease, and neurological disease can occur, especially with chronic infections	*Bartonella* spp. ELISA (Lappin et al. 2008)
Feline Immundeficiency Virus	Lentivirus	Direct contact	Immunosuppression after multiple years of infection or no clinical signs	FIV Western Blot (Franklin et al. 2007)
*Toxoplasma gondii*	Protozoan	Ingestion of intermediate host or oocysts from environment	Limited symptoms in healthy cats	*Toxoplasma gondii* ELISA for both IgM and IgG antibodies (Vollaire et al. 2005)

In this study we utilized a unique dataset of pathogen exposure among domestic and non-domestic felids across multiple geographic locations in California and Colorado (Figure 1). The three different pathogens evaluated in this study represent a broad range of taxonomic groups (viral, protozoal, bacterial) and diverse transmission mechanisms (direct contact, environmental/ingestion, vector-borne; Table 1). We evaluated how species, age and geographic location predicted seroprevalence. Comparison of pathogens also provides insight into routes by which pathogens can invade and move within and between species. Accordingly, *a priori* hypotheses based on modes of transmission, differ in their predictions for each pathogen, host species and site. *Bartonella* spp. seroprevalence is hypothesized to be more common in areas with heightened vector activity (*i.e.* moist and warm climates). Feline Immunodeficiency Virus strains are primarily species-specific [18], [20] and so it is hypothesized that seroprevalence will differ among felid species, reflecting species-specific densities and contact rates. Since FIV is spread through direct contact, especially aggressive interactions [34], [35], it is also likely to be more common in males. *Toxoplasma gondii* is ubiquitous in the environment and is often transmitted through consumption of infected prey [27], [28], [29]. Therefore, different locations, species, and sexes are hypothesized to similar exposure levels. Age is believed to be a factor related to exposure status for all three pathogens, with older animals more likely to have experienced infection because of increased exposure risk over time.

## Results

### Seroprevalence

**Figure 2:**
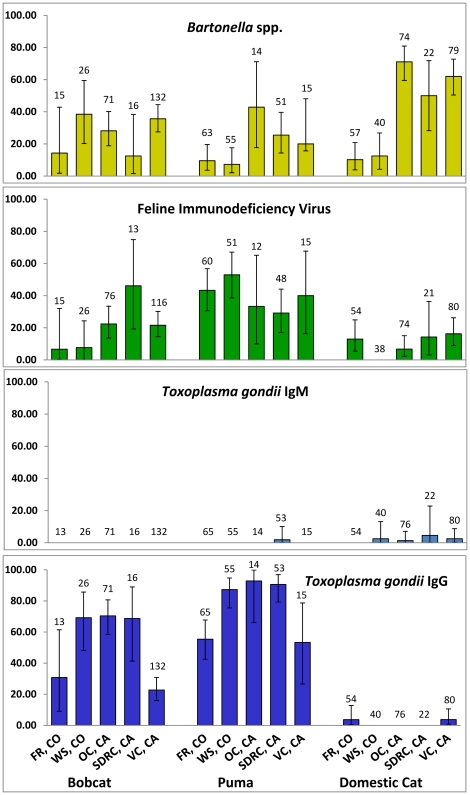
Seroprevalence, with bars representing 95% confidence intervals, of *Bartonella* spp., FIV, and *T. gondii* IgG for domestic cats, bobcats, and pumas at all study locations (FR  =  Front Range, CO; WS  =  Western Slope, CO; OC  =  Orange County, CA; SDRC  =  San Diego/Riverside Counties, CA; VC  =  Ventura County, CA). Sample sizes are listed above columns.

Average seroprevalence revealed general trends in pathogen exposure both within and across felid species and locations (Figure 2). *Bartonella* spp. seroprevalence varied considerably, but in almost all cases, *Bartonella* spp. seroprevalence was higher in California than in Colorado (Figure 2). For domestic cats in California, *Bartonella* sp. seroprevalence positively reflected proximity of sampling locations to large urban areas. Domestic cats sampled in Orange County, CA and Ventura County, CA, directly south and north, respectively, of the major metropolitan area of Los Angeles, had the highest *Bartonella* spp. seroprevalence rates of all locations and species (Figure 1, Figure 2). Feline Immunodeficiency Virus seroprevalence was higher in non-domestic felids compared to domestic felids. *Toxoplasma gondii* IgM seroprevalence (indicative of recent infection) was low in all species and locations, whereas *T. gondii* IgG seroprevalence was higher in non-domestic felids compared to domestic felids (Figure 2).

**Figure 3:**
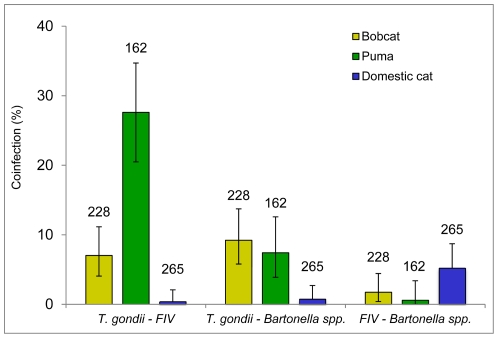
Coinfection rates, with bars representing 95% confidence intervals, of FIV/*T. gondii* IgG coinfection, *T. gondii* IgG*/Bartonella* spp. coinfection, and FIV/*Bartonella* spp. coinfection, for bobcats (n = 228), pumas (n = 162), and domestic cats (n = 265).

The most common pathogen co-occurrence was identified in pumas infected with both *T. gondii* and FIV; this condition was much less common in domestic cats (Figure 3). Bobcats had the highest occurrence of having been exposed to both *T. gondii* and *Bartonella* spp., although puma *T. gondii* and *Bartonella* spp. exposure levels were similar, while domestic cat levels were much lower (Figure 3). Interestingly, dual *Bartonella* spp. and FIV exposure was relatively rare in the non-domestic species, but were the most common co-occurring pathogen exposures recorded in domestic cats (Figure 3). Only 3.5% of bobcats (95% CI 1.5–6.8), 0% of domestic cats (95% CI 0.0–1.4), and 4.3% (95% CI 1.7–8.7) of pumas had evidence of exposure to all three pathogens (Figure 3).

### Association with specific variables

Age, location, and species were associated with *Bartonella* spp. IgG antibody presence (Table 2, Nagelkerke pseudo-R^2^ = 0.22). Sex was also included in another model with slightly less support (Table 2). Odds ratios for the age effect revealed that the odds of adult animals being seropositive were 1.6 times as large than the odds of young animals being seropositive, although adjusted 95% confidence intervals (CI) showed this to be a borderline effect (Table 3). Domestic cats were more likely to be seropositive when compared to both pumas and bobcats, while bobcats and pumas had similar exposure rates (Table 3). Overall, the three California locations had substantially and consistently higher *Bartonella* spp. seroprevalence rates when compared to Colorado locations (Table 3). This pattern was consistent for domestic cats and puma, but not for bobcats (Figure 2). Overall, the similar seroprevalence of all three California locations to each other, in concert with the two Colorado locations having similar seroprevalence rates, underscores the association of *Bartonella* spp. exposure to broad-scale geographic location. In addition, *Bartonella* spp. was highest at the two most urban study locations, Orange County, CA and Ventura County, CA, particularly for domestic cats (Figure 2, Table 3).

**Table 2:** Best supported models (ΔAICc <2) of seroprevalence for the three pathogens.

Pathogen	Best-Supported Models	K	−2 Log Likeli-hood	AICc	Δ	*w*
*Bartonella* spp.	age+location+species	8	702.35	718.58	0	0.59
	age+location+sex+species	9	702.18	720.18	1.6	0.26
Feline Immunodeficiency Virus	age+sex+species	5	592.37	602.47	0	0.68
	age+species	4	595.93	603.99	1.52	0.32
*Toxoplasma gondii* IgG	age+location+species	8	451.44	467.67	0	0.73
	age +location+ sex +species	9	451.39	469.68	2.01	0.27

**Table 3:** Odds ratios and adjusted 95% confidence limits for parameters from best supported models.

Pathogen	Parameter	Comparison	Odds Ratio	Adjusted 95% Confidence Limits
*Bartonella* spp.				
	Age	Adult vs. Young	1.6	1.0–2.5
	Species	Domestic vs. Bobcat	3.33	2.0–5.0
		Domestic vs. Puma	2.6	1.3–4.9
		Puma vs. Bobcat	1.1	0.5–2.5
	Location	Orange County, CA vs. Front Range, CO	10	5.0–33.3
		Orange Couny, CA vs. Western Slope, CO	5.3	2.2–12.5
		Orange Couny, CA vs. San Diego/Riverside Counties, CA	1.8	0.7–4.3
		Orange County, CA vs. Ventura County, CA	1.2	0.6–2.3
		San Diego/Riverside Counties, CA vs. Front Range, CO	10	2.0–16.6
		San Diego/Riverside Counties, CA vs. Western Slope, CO	2	1.1–7.8
		Ventura County, CA vs. San Diego/Riverside Counties, CA	1.6	0.6–5.0
		Ventura County, CA vs. Western Slope CO	2.3	1.7–10.1
		Ventura County, CA vs. Front Range, CO	10	3.3–25.0
		Western Slope, CO vs. Front Range, CO	2	0.5–6.2
FIV				
	Age	Adult vs. Young	2.8	1.6–5.0
	Species	Bobcat vs. Domestic	2.5	1.3–4.7
		Puma vs. Bobcat	3.3	2.0–10.0
		Puma vs. Domestic	8.3	5–16.6
	Sex	Male vs. Female	1.6	1.0–2.5
*Toxoplasma gondii*				
	Age	Adult vs. Young	4.4	2.5–7.8
	Species	Bobcat vs. Domestic	72.2	23.1–225.5
		Puma vs. Bobcat	10	2.5–12.5
		Puma vs. Domestic	333.3	111.1–1000
	Location	Orange County, CA vs. Front Range, CO	7.6	2.5–25
		Orange Couny, CA vs. Western Slope, CO	1.4	0.4–4.3
		Orange Couny, CA vs. San Diego/Riverside Counties, CA	1	0.2–3.5
		Orange County, CA vs. Ventrua County, CA	2.8	1.1–6.6
		San Diego/Riverside Counties, CA vs. Front Range, CO	7.6	2.5–33.3
		San Diego/Riverside Counties, CA vs. Ventura County, CA	2.8	0.8–9.5
		San Diego/Riverside Counties, CA vs. Western Slope, CO	1.4	0.4–5.1
		Ventura County, CA vs. Front Range, CO	3.3	0.8–8.3
		Western Slope, CO vs. Front Range, CO	5	1.6–16.6
		Western Slope CO vs. Ventura County, CA	2	0.7–10.0

Feline Immunodeficiency Virus infection models (Table 2, Nagelkerke pseudo-R^2^ = 0.18) consisted of age, sex, and species. As hypothesized, older animals of all species were more likely to be infected and males were 1.6 times more than the odds of females to be infected with FIV, although 95% CI showed this effect to be subtle (Table 3). Domestic cats had consistently lower prevalence rates compared to the non-domestic species (Figure 2, Table 3); the odds of being FIV positive for pumas were 8 times as large as the odds of domestic cats being FIV positive (Table 3). Contrary to *Bartonella* spp., FIV infection was not associated with location, indicating that broad-scale geographic location does not explain differences in FIV prevalence.

Prevalence of *T. gondii* IgG was predicted by age, location, and species (Table 2, Nagelkerke pseudo-R^2^ = 0.63). Again, as hypothesized, older adult animals were more likely to have been exposed to *T. gondii* compared to younger animals (Table 3). Geographic seroprevalence of *T. gondii* antibodies was not as consistent across locations as *Bartonella* spp. exposure. In Colorado, *T. gondii* seroprevalence was higher in the rural Western Slope location compared to the urban Front Range location (Table 3). In California locations, odds ratios and 95% CI for *T. gondii* exposure often overlapped (Table 3), suggesting similar exposure rates (Figure 2). Pumas and bobcats had substantially higher exposure rates to *T. gondii* IgG compared to domestic cats (Table 3), generating extremely large odds ratios because of the low domestic cat seroprevalence. Additionally, for pumas, the odds of being seropositive were 10 times as large as the odds for a bobcat to be seropositive, with seroprevalence in some areas approaching 100% (Figure 2, Table 3).

## Discussion

The fundamentals of zoonotic disease ecology are often poorly understood despite the fact that they can have serious public health consequences and are emerging with alarming frequency [3], [36]. In addition, threatened species, as well as overall biodiversity, can be negatively impacted by disease [1], [2], [37], [38]. This study incorporated data collected over a ten year period on 791 pumas, bobcats, and domestic cats, sampled across 5 study areas that varied in both ecosystem characteristics and degree of urbanization (Figure 1). Data provide new and unanticipated findings about the distribution of three pathogens capable of infecting and being transmitted among three felid species whose ranges overlap, particularly along urban edges. This study revealed specific associations between variables of interest and exposure to pathogens, and found that transmission route was consistently associated with the variables driving exposure. Results have implications for the routes in which emerging or invading pathogens could move within and between these species.

*Bartonella* spp. exposure, as predicted, was generally higher in California locations when compared to Colorado locations. The initial prediction was based on the known association between *C. felis* and *Bartonella* spp. known to infect cats, coupled with data on the relationship between flea distributions and climate [39]. Potential arthropod vectors occur in higher numbers in regions with warmer temperatures and higher humidity[39] and these climate differences may drive the higher exposure levels seen in California felids. Domestic cats in California had substantially higher *Bartonella* spp. exposure than non-domestic cats which could be related to high domestic cat densities in urban areas, leading to locally amplified flea populations and increased transmission opportunities. Published densities of domestic cats adjacent to our Ventura County site are much greater (2–3 orders of magnitude) than non-domestic cats [8], [11], [40], [41], [42], a situation that is potentially similar in other urban sites as well. Previous research has demonstrated that *Bartonella* seroprevalence in domestic cats in the Los Angeles region is even higher than predicted based on flea prevalence estimates [39], and while flea and host species richness is higher in non-urban areas, flea infestation was higher in urban/disturbed sites with low vector richness [43]. In support of these previous findings, we observed higher *Bartonella* spp. seroprevalence among domestic cat populations nearer large urban centers in California (*i.e.* Orange County, CA and Ventura County, CA). Despite vector research that supports the felid seroprevalence patterns reported here, flea data was not collected during the course of this study. Future flea collections, along with sequencing of bacterial isolates from fleas and feline blood, are needed to definitively relate host seroprevalence to differences in vector abundance and distribution. Additional vectors, such as ticks, should be examined as well based on evidence that other vectors could be involved in *Bartonella* spp. transmission [12], [13]. These more detailed data might also help reveal why bobcats from the rural Colorado Western Slope had substantially higher *Bartonella* spp. exposure when compared to sympatric pumas and domestic cats.

*Toxoplasma gondii* has received substantial attention because it is a ubiquitous pathogen with significant zoonotic potential [31], [44]. Studies have primarily focused on human infection and disease, but links to marine mammal population declines have also been documented [31]. The primary factors associated with *T. gondii* seroprevalence in this study were the same factors associated with *Bartonella* spp. exposure: age, location, and species. The similarity between the models for the two pathogens may be related to the fact that exposure for both is typically indirect (*i.e.*, vector transmission for *Bartonella* and ingestion of infected prey or environmental contact for *T. gondii*) rather than direct contact between conspecifics. Despite both pathogens having similar predictors in the final models, the overall seroprevalence patterns differed. Pumas had higher *T. gondii* seroprevalence across all regions compared to bobcats, suggesting pumas have increased exposure to the pathogen. This could be reflective of factors including: (1) larger home range and spatial scales of pumas, resulting in increased exposures; (2) a larger dietary intake, resulting in a greater opportunity for ingestion of prey species with encysted toxoplasmosis intermediate forms; (3) a diet that consists of prey species with higher *T. gondii* exposure; or, (4) increased susceptibility of pumas to *T. gondii* infection. Dual exposure to both *T. gondii* and FIV was substantially higher in pumas compared to both bobcats and domestic cats. This is likely simply related to both *T. gondii* and FIV being independently common and so they are more likely to co-occur, but is also possible that there may be an interaction between the two pathogens that increases opportunities for infection, or that some aspects of puma behavior are “risky,” leading to increased exposure to both pathogens.

Domestic cats, despite the attention they generate as a source of *T. gondii* infections in households, had extremely low seroprevalence overall to this parasite. This is supported by data from several countries that show human exposure to *T. gondii* is often related to the meat supply and meat consumption, rather than exposure to domestic cats [45], and is likely reflective of the limited ingestion of intermediate host species around urban areas by domestic cats versus their free-ranging relatives. It is possible that stray and/or feral domestic cat populations consisted of disproportionately younger animals, and that this contributed to the lower than expected *T. gondii* seroprevalence reported here [46], [47]. The higher seroprevalence in non-domestic felids suggests that *T. gondii* could represent a scenario where disease exposure is higher in undeveloped areas and along an urban edge versus developed, urban areas. A recent study modeling *T. gondii* transmission via environmental contact or intermediate host suggested that urban transmission was more dependent upon environmental exposure whereas suburban/rural spread was more dependent upon ingestion of intermediate hosts [48]. A similar model of *Baylisascaris procyonis* transmission in raccoons (*Procyon lotor*) also predicted differences in intermediate host exposures in urban versus rural landscapes [49]. The highest *T. gondii* seroprevalence levels seen in this study were in non-domestic felids from rural study areas, although substantial exposure was also seen in urban Orange County, CA pumas and bobcats. Our data would therefore support a robust means of sylvatic infection (resulting in high infection rates of pumas and bobcats outside of urban centers) with a less efficient exposure in urban settings (as reflected by low exposure in feral domestic cats). The broad spatial scales examined confound a detailed analysis of pathogen exposure in relation to urbanization and warrant further study on finer spatial scales.

In contrast to bartonellosis and toxoplasmosis, FIV is transmitted horizontally through direct contact, especially aggressive interactions or mating. Location was associated with *Bartonella* spp. and *T. gondii* seroprevalence, but was not a predictor of FIV exposure in any species. This does not necessarily rule out spatial heterogeneity in FIV exposure or focal regions where FIV is more prevalent, but such associations were not detected here. The FIV model explained less variation than the models for the other two pathogens and it is possible that this unexplained variation could be related to localized clusters that were not accounted for in the analysis. Future analysis on finer spatial scales may help to pinpoint additional sources of variation.

While not a strong predictor, males were more likely to be infected with FIV than females in all three species. This pattern is consistent with previous research [35], which has related the frequency of aggressive interactions among male felids to increased FIV transmission opportunities. Species-specificity of FIVs has been well-documented [18], [20], [50] and we noted substantial differences in FIV seroprevalence among bobcats, pumas, and domestic cats. Greater genetic diversity among non-domestic FIV strains suggests that domestic cat FIV emerged relatively recently whereas FIV of wild felids has been established for a longer period of time [18], [20]. In this study, FIV exposure was consistently lower in domestic cats compared to non-domestics, which is consistent with other serosurveys of domestic cats in the US [20]. While domestic cat sample collection focused on feral and stray animals, it is likely that some domestic cats were “owned” at some point and those conditions, which could include neutering and some degree of isolation from other cats, resulted in decreased FIV exposure. Interestingly, the most common pathogen combination in domestic cats was exposure to both FIV and *Bartonella* spp. While previous research has found an association between *Bartonella* spp. in felids and another feline pathogen, Feline Leukemia Virus [51], it is also possible that the association found here simply reflects similar risk factors.

In summary, this analysis suggests that environment, species, and individual behaviors are important factors in disease occurrence, and that anthropogenic influences may alter pathogen structure in wild populations. Conversely, wild populations of felids appear to be important reservoirs for some highly prevalent human diseases. Diseases which spread within species – such as FIV – are less likely to be influenced by geographic location and are more likely dependent upon individual behaviors and intra-specific encounters. Pathogens that are spread by vectors, like *Bartonella* spp., are more likely to occur in regions supporting vector success and vector exposure to the pathogen, though domesticated animals may serve as focal bioaccumulators that could impact prevalence among susceptible wildlife. Pathogens transmitted by environmental contamination or ingestion of infected prey (i.e. toxoplasmosis) can have broad regional associations, and our studies provide evidence that *T. gondii* exposure is substantial in rural settings, and therefore wildlife may serve as reservoirs for domestic animals and/or human toxoplasmosis when at the urban-wildland interface.

Domestic cat densities are higher in urban areas [52], while puma and bobcat populations can decrease as a consequence of urbanization and habitat isolation [53]. Wild felids isolated by habitat fragmentation exhibit “home-range pile-up” [10] and the potential for increased contact rates with conspecifics. Contact between domestic cats and non-domestic felids can lead to cross-species FIV transmission events, such as has apparently occurred with feline leukemia virus transmission between domestic cats and pumas in Florida [54]. Additionally, recent spatial analyses have suggested that landscape features, and in particular roads, could impact FIV infections in pumas [55]. Additional studies will focus on use of chronic pathogen genetic signatures to trace wildlife movement in fragmented landscapes and predictive modeling for fine scale analysis of diseases in carnivores impacted by human development.

## Materials and Methods

### Ethics Statement

Animal handling and capture was approved by the Colorado State University Animal Care and Use Committee, protocol #11-2453A, and procedures underwent extensive review and discussion in order to institute practices that minimized suffering.

### Sample collection and processing

Opportunistic samples from bobcats and pumas were obtained from collaborators performing ongoing biology and ecology research on bobcats and puma. Samples from domestic cats were collected from free-ranging domestic cats on admission to shelters, or through domestic cat trap, neuter, release programs. Pumas, bobcats, and domestic cats were sympatrically sampled from study sites that encompassed both urban and rural locations (Figure 1, urbanization information derived from 2006 National Land Cover Database and presented using ArcGIS 9.3.1, ESRI 2010). Three California study sites included Ventura County, Orange County, and the eastern portion of San Diego County and Riverside County. In Colorado, samples were collected across a large part of the Western Slope and the northern portion of the Front Range (Figure 1).

A majority of samples were collected between 2000 and 2010, but 25 samples from Ventura County were collected in the late 1990 s. These large temporal and spatial scales allowed for collection of unprecedented sample sizes and provided information on essentially chronic infectious diseases that are not thought to be governed by epizootic dynamics. Bobcats and pumas were captured using a variety of tranquilizers/sedatives [7], [56], sampled, and released with the permission of cooperating agencies. Domestic cats were sampled opportunistically during veterinary examinations. Animal handling and capture was approved by the Colorado State University Animal Care and Use Committee, protocol #11-2453A, and procedures underwent extensive review and discussion in order to institute practices that minimized suffering. Animal age category was estimated in the field based on size, weight, and dental wear [7]. Blood from live animals was collected in ethylenediaminetetraacetic acid (EDTA) or serum-separating tubes, processed according to protocol [57], and stored at −80°C.

### Assays

*Toxoplasma gondii* and *Bartonella* spp. assays were carried out at the Center for Companion Animals Studies at Colorado State University (CSU; Fort Collins, Colorado). Feline Immunodeficiency Virus detection was completed in the Feline Retrovirus Research Laboratory in the Microbiology, Immunology, and Pathology Department at CSU. Serum samples were analyzed for FIV, *Bartonella* spp., and *T. gondii* (Table 1). Assays were performed and interpreted following the standard operating procedures for individual laboratories as previously described [28], [57], [58] and briefly outlined below (Table 1).

Feline Immunodeficiency Virus infection in bobcats and pumas was determined using western blot analysis designed to detect puma lentivirus (PLV) 1695, a puma specific FIV strain isolated from a puma in British Columbia. PLV-1695 western blot analysis has previously been shown to be the most sensitive detection system for seropositivity for bobcat and puma FIV strains [57]. Viral stocks were grown in domestic cat Mya-1 cell lines [59], and viral proteins were isolated as previously described [60]. Antigens were prepared from viral cultures and 50 mg of viral antigen was run on a 12% polyacrylamide gel. The antigen was subsequently transferred to an Immun-Blot™ polyvinylidene difluoride membrane (Bio Rad Laboratories) and analyzed as described in Franklin *et al*. [57]. Serum or plasma samples were diluted 1∶50 in phosphate buffered saline. Positive-control sera (cat sera from an experimentally FIV infected domestic cat) and negative cat sera were also diluted 1∶50. Western blotting was performed as previously described [57]. Reaction strength was assessed visually and was scored depending on the affinity of the antibody for the p24 gag protein: 0, negative; 1, equivocal; 2, positive; 3, strongly positive. Samples scored as 1 were either re-tested or conservatively recorded as negative.

Feline Immunodeficiency Virus infection in domestic cats was determined by the above described Western Blot (but using purified and pelleted FIV from an experimentally infected domestic cat, 2104) and by ELISA (enzyme-linked immunosorbent assay) following previously established methods [61]. Briefly, ELISA plates were coated (100 µL/well) with whole-pelleted domestic cat FIV (from an experimentally infected domestic cat, 2104) diluted to 750 ng/100 µL in 0.01 M borate buffer (20 g/L borax, 1 g/L boric acid) containing 0.5% deoxycholic acid and incubated overnight at 4°C. Plates were then washed five times in NTE buffer (0.5 M NaCl, 0.05 M Tris, 0.001 M EDTA) containing 0.2% Tween-20 and blocked (200 µL/well) with NTE buffer containing 2% BSA at 4°C for 24 hours. Following blocking, plates were washed (as above) and feline serum samples were added in duplicate, along with positive and negative control sera. All sera (100 µL/well) were first diluted 1∶100 in ELISA buffer (NTE with 2% BSA, 5% FCS and 0.5% Tx-100) and incubated for 60 minutes at room temperature. Plates were then washed and incubated (100 µL/well) with goat anti-cat peroxidase conjugate (Cappel) and diluted 1∶2000 in ELISA buffer for 60 minutes at room temperature. Plates were washed again and incubated with TMB (100 µL/well) for 15 minutes at room temperature. The reaction was then stopped with 2.5 N H_2_SO_4_ (50 µL/well) and optical density read at 450 nm. Samples were considered to be positive at optical density ≥0.35 nm, as validated by Combo SNAP™ test (Idexx Laboratories, Westbrook, Maine, USA) and immunoblot.

Serum was analyzed for evidence of antibodies to *Bartonella* spp., and *T. gondii* using previously developed ELISA protocols [28], [47]. The *T. gondii* specific ELISA detected both immunoglobulin M (IgM), which indicates recent infections and is usually detectable ≤ 16 weeks after initial exposure, as well as immunoglobulin G (IgG) [62], which is detectable for ≥52 weeks after infection [63]. Two measures of *T. gondii*, IgM and IgG, are often reported because of the potential for zoonotic transmission in recently exposed (*i.e.* IgM positive) animals. The *Bartonella* spp. ELISA used *B. henselae* as the antigen source and detects IgG antibodies for *B. henselae, B. clarridgeiae*, and *B. koehlerae* [14]. The lowest positive titer for both pathogen assays was 1∶64.

### Data Analyses

#### Prevalence estimation

Samples were analyzed from 791 individual animals, although limited sample volume prevented some animals from being screened for all three pathogens, resulting in slightly different sample sizes (Figure 2). Missing location and categorical data for some samples also precluded the inclusion of all samples in Figure 1 and in logistic regression models. Recaptured animals were only counted once in seroprevalence calculations and were considered positive if any sampling time point was positive. Describing pathogen exposure was a primary goal of this analysis and this prevented animals with multiple recaptures from artificially affecting results. Mean seroprevalence and associated 95% confidence intervals were calculated using a binomial distribution for each species and location.

#### Determinants of exposure to infectious agents

The best model to describe the association between seroprevalence for each pathogen and biologically relevant independent variables was determined using the small sample size corrected Akaike's information criterion (AICc) for model selection [64]. All variables were categorical and included location (Western Slope, CO; Front Range, CO; San Diego/Riverside Counties, CA; Orange County, CA), species (bobcat, puma, domestic cat), age (adult animals ≥2years; young animals 6 months - 2 years), and sex (male, female) of sample animals. Interaction effects were not explored because of sample size limitations that arise when overly partitioning binomial data. *A priori* hypotheses determined the factors to be included in each initial set of models, and models for each pathogen were tested with all combinations of the four factors (location, species, age, sex). The strongest models with AICc Δ values <2 were identified and reported along with Nagelkerke pseudo R^2^ [64], [65].

Associations between model-selected risk factors and exposure to each of the three pathogens – *Bartonella* spp., FIV, and *T. gondii* IgG – were analyzed with SAS version 9.1. Analyses used a logistic link function and binary error using antibody presence (positive vs. negative) for each pathogen as the outcome variable. Pairwise differences in the least square means were analyzed using t tests with a Tukey-Kramer adjustment for multiple comparisons. Odds ratios were reported to provide a relative magnitude of the association of infection with the determinants. Adjusted 95% confidence intervals were calculated to simultaneously allow for the effect of the other predictors and are reported as well.
